# Falling down the biological rabbit hole: Epstein-Barr virus, biography, and multiple sclerosis

**DOI:** 10.1172/JCI164141

**Published:** 2022-09-01

**Authors:** Ralph I. Horwitz, Allison Hayes-Conroy, Burton H. Singer, Mark R. Cullen, Kimberly Badal, Ida Sim

**Affiliations:** 1 Lewis Katz School of Medicine and; 2 Department of Geography and Urban Studies, Temple University, Philadelphia, Pennsylvania, USA.; 3 Emerging Pathogens Institute, University of Florida, Gainesville, Florida, USA.; 4 Stanford University, Stanford, California, USA.; 5 Helen Diller Comprehensive Cancer Centre and; 6 Division of General Internal Medicine and Computational Precision Health, UCSF, San Francisco, California, USA.

## Introduction

A recent research report in *Science* by Bjornevik et al. tested the hypothesis that multiple sclerosis (MS) is caused by Epstein-Barr virus (EBV) in a cohort of more than ten million adults on active duty in the US military during a 20-year period (1993–2013) (1). The authors reported that individuals who had prior EBV infection were 24 times more likely to develop MS than noninfected persons.

The findings appear to confirm a longstanding suspicion linking EBV to MS and led many to call for an EBV vaccine to prevent MS. A closer examination of the article, however, indicates the analysis was incomplete and misrepresents the data. In fact, a strong association between EBV and MS was present only for those with recent infection occurring during active-duty military service.

## EBV and MS: role of active-duty service

In the Bjornevik study, the investigators took advantage of a program in which the US Department of Defense stored serum samples from active-duty members of the military on entry and biennially afterward for the purpose of screening for HIV. Samples from 801 donors with MS (cases) were matched to those from 1,566 individuals without MS (controls) based on sex, age, race/ethnicity, branch of service, and sample date (1).

Eight hundred of the 801 cases (99.9%) were EBV positive in the most recent samples taken before MS onset, compared with 1,520 of the controls (97%) (OR 24.2, *P* < 0.001). However, these 801 cases differed in one critical aspect: one group was seropositive at entry into the military, with evidence for remote infection before active service; another group was seronegative at entry and became infected with EBV during military service. Looking only at the group that was seropositive at entry, the OR for EBV and MS was only 1.6 (*P* < 0.05), a far cry from 24.2; 766 of 801 cases were EBV positive (95%) compared with 1,459 of 1,566 controls (93%). It was the second group — those who became infected with EBV during military service — that demonstrated the stunningly high OR. This group consisted of 35 cases and 107 controls who were EBV negative at baseline. During the follow-up, all but one of the 35 cases (97%) but 61 of the 107 negative controls (57%), seroconverted, for an OR of 25.6 (*P* < 0.001).

## Biosocial pathways to MS

Rather than constituting a simpler top-line story that EBV infection confers a 24-fold risk of MS, the findings indicate that on entry into the military, remote infection with EBV had only a weak association with MS. A strong association between EBV and MS was present only for those with recent infection occurring during active-duty military service.

What happened during military service to the set of 35 cases and 107 controls who were EBV negative at baseline that could account for the 24-fold risk? Here we must resist the temptation to explore only biological explanations, but rather consider biosocial pathways that are mediated through exposures, behaviors (especially those imposed by work), and the emotional affect that results from our relationship with people and world around us (2). With this framework, the potential cofactors might be biological (including genetic susceptibility and the microbiome), environmental (including toxins, changes in latitude), or related to stressful or traumatic experiences. Did case and control individuals differ in deployment assignments? Did different deployments lead to differences in diets and the individual’s microbiome or to combat or noncombat stresses or trauma, either personal or witnessed by comrades?

Resolving these and other considerations in the lived experience of the service members is crucial, since there is accumulating evidence that social and psychological stress and life disruptions can contribute to the likelihood of both acquiring infection and developing postinfectious sequelae. Consider the mechanisms that link biography to respiratory infection or vaccine effectiveness. Studies using a viral challenge model to understand how affective factors influence the risk of infection with cold or influenza viruses revealed an increased risk of infection in patients with high levels of psychosocial stress (3, 4). Similarly, stress, depression, and loneliness are associated with less-robust immune responses to vaccination and alter the prevalence and severity of vaccine side effects (5).

There is already a growing evidence base indicating that the lived experience of military service does indeed influence susceptibility to disease. Studies of cadets at the US Military Academy at West Point provided strong evidence that psychological stressors modulated the steady-state expression of latent EBV, resulting in reactivation of latent virus (6). The investigators assessed serum antibody levels in response to three latent herpesviruses — EBV, HSV-1, and human herpesvirus 6 — in the cadets at four different times. The psychological tests included the NEO Personality Inventory, Health Hardiness Questionnaire, Social Support Questionnaire, and Perceived Stress Questionnaire. Serum samples collected during final academic examinations showed significant antibody elevations, and the results were consistent with those of other studies in participants experiencing psychological stress (6).

There are also numerous lines of evidence that link stress-related conditions to autoimmune disease in both civilians and military populations. A recent cohort study of Swedish civilians with stress-related disorders reported an elevated risk of autoimmune disease, and individuals with preexisting diagnosed post-traumatic stress disorder (PTSD) had an increased risk for multiple autoimmune disorders (7). In a cohort study of US veterans previously deployed to Iraq or Afghanistan, those who had sought care for PTSD had increased risks for rheumatoid arthritis (RA), systemic lupus erythematosus (SLE), inflammatory bowel disease (IBD), and MS (8).

Finally, and most suggestively, the Millennium Cohort Study of US service members was designed to examine prospectively the association between a stress-related disorder, PTSD, and the risk of several of the more common autoimmune disorders, including RA, SLE, IBD, and MS. In an analysis of 120,572 active-duty soldiers, those with PTSD reported more combat exposure as well as physical and sexual trauma. In a mean of 5.2 years of follow up, 864 participants developed new-onset autoimmune disease. Among those with PTSD, the risk for any of the autoimmune diseases was 60% higher, and it was highest for MS (8).

## Biosocial mechanisms: the next research frontier

A recent review of the biological mechanisms that would explain how EBV might lead to MS concluded by noting that “[p]lausible biological mechanisms for EBV’s pathogenic role in MS have now been elucidated, but a universal unifying mechanism has not yet been identified” (9). There is good reason to believe that biology alone is unable to provide such a unifying mechanism, and there is a need to elucidate the “biosocial pathogenesis” by integrating biology and biography — the individual’s lived experience — in order to understand the pathway between EBV infection and MS (10).

There is now ripe opportunity for a deeper exploration of the biosocial pathogenesis of EBV and MS (10). The study design employed by Bjornevik et al. was well suited for the serological analysis they performed. Additional quantitative analyses that would provide further insights should be possible. Determining the timing of infection after seroconversion would help to identify potential windows of differential vulnerability. Analyzing the entire active duty cohort to estimate the cumulative incidence of MS would suggest whether active military members have an unusually high rate of occurrence of MS compared with civilian populations.

Even more promising, the Department of Defense data used by Bjornevik et al. could be combined with the extensive information that the military maintains on active-duty personnel. This analysis might begin with identification of major social and behavioral features suggested by the National Academy of Medicine (11) and other investigators (12), but tailored to this situation — such as locations and types of deployments; stressful events including active military engagement, or loss of friends or colleagues during battle; PTSD, anxiety, or depression; and many more factors. Allostatic load, a physiological measure of the cumulative burden of stress on the body, could also be assessed by biomedical markers of physiological dysregulation. These features can be used to model the risk of MS after EBV seroconversion with a biosocial analytical structure.

Numerous commentators have suggested that what is needed now is an EBV vaccine to prevent MS. We caution against falling down this biological rabbit hole. Although the data indicate that EBV infection may be a necessary precondition for MS, there is much we still do not know about the biological and biographical triggers of MS. Fortunately, the military has extensive information on the lived experience of active-duty military personnel that may offer breakthrough insights into the relationship between EBV and the biosocial pathogenesis of MS.

## Version 1 09/01/2022

Electronic publication
